# Human mesenchymal stem cells labelled with dye-loaded amorphous silica nanoparticles: long-term biosafety, stemness preservation and traceability in the beating heart

**DOI:** 10.1186/s12951-015-0141-1

**Published:** 2015-10-29

**Authors:** Clara Gallina, Tânia Capelôa, Silvia Saviozzi, Lisa Accomasso, Federico Catalano, Francesca Tullio, Gianmario Martra, Claudia Penna, Pasquale Pagliaro, Valentina Turinetto, Claudia Giachino

**Affiliations:** Department of Clinical and Biological Sciences, University of Turin, 10, Regione Gonzole, CAP 10043 Orbassano, TO Italy; Department of Chemistry, Interdepartmental Centre “Nanostructured Interfaces and Surfaces”, University of Turin, 7, Via P. Giuria, CAP 10125 Turin, Italy

**Keywords:** Mesenchymal stem cells, Silica nanoparticles, Toxicity, Stem cell tracking, Heart

## Abstract

**Background:**

Treatment of myocardial infarction with mesenchymal stem cells (MSCs) has proven beneficial effects in both animal and clinical studies. Engineered silica nanoparticles (SiO_2_-NPs) have been extensively used as contrast agents in regenerative medicine, due to their resistance to degradation and ease of functionalization. However, there are still controversies on their effective biosafety on cellular systems. In this perspective, the aims of the present study are: 1) to deeply investigate the impact of amorphous 50 nm SiO_2_-NPs on viability and function of human bone marrow-derived MSCs (hMSCs); 2) to optimize a protocol of harmless hMSCs labelling and test its feasibility in a beating heart model.

**Results:**

Optimal cell labelling is obtained after 16 h exposure of hMSCs to fluorescent 50 nm SiO_2_-NPs (50 µg mL^−1^); interestingly, lysosomal activation consequent to NPs storage is not associated to oxidative stress. During prolonged culture hMSCs do not undergo cyto- or genotoxicity, preserve their proliferative potential and their stemness/differentiation properties. Finally, the bright fluorescence emitted by internalized SiO_2_-NPs allows both clear visualization of hMSCs in normal and infarcted rat hearts and ultrastructural analysis of cell engraftment inside myocardial tissue.

**Conclusions:**

Overall, 50 nm SiO_2_-NPs display elevated compatibility with hMSCs in terms of lack of cyto- and genotoxicity and maintenance of important features of these cells. The demonstrated biosafety, combined with proper cell labelling and visualization in histological sections, make these SiO_2_-NPs optimal candidates for the purpose of stem cell tracking inside heart tissue.

**Electronic supplementary material:**

The online version of this article (doi:10.1186/s12951-015-0141-1) contains supplementary material, which is available to authorized users.

## Background

Acute myocardial infarction (MI) is a pathological condition that often results in large-scale loss of cardiac muscle. Interestingly, several works documented that ischemic cardiac injury is able to stimulate the recruitment into the myocardium of circulating stem cells (SCs) [1, 2]. In the context of MI therapy, a vast number of preclinical studies observed significant cardiac improvement after mesenchymal SCs (MSCs) transplantation [3, 4] and regenerative therapy using MSCs has been recently detailed in several clinical trials [5–7]. However, drawbacks reside on the complexity to accomplish proper cell delivery and in the still poor understanding of the exact mechanisms at the base of cell distribution inside the injured tissue. The development of safe imaging techniques is therefore of great interest to allow long-term analysis of cell survival, migration and engraftment, facilitating the understanding of treatment outcomes [8, 9]. Several engineered nanoparticles (NPs), defined as ultrafine objects with a size less than 100 nm [10], have already been tested in preclinical studies to follow MSCs inside the cardiac milieu [11, 12]. Among them, silica NPs (SiO_2_-NPs) possess unique properties of biocompatibility, stability over time and easily adjustable properties, for example size, morphology, porosity and surface chemistry [13].

To date, SiO_2_-NPs have already been used in animal studies to follow the fate of MSCs inside the host [14, 15]. However, despite promising results, there is considerable controversy about the safety of silica nanomaterials on cellular systems, mainly due to differences in the synthesis method, size and shape of the platforms [16, 17]. Therefore, for the safe application of SiO_2_-NPs for SCs tracking it is crucial to conduct systematic studies to assess their toxicological profile in terms of potential interference with self-renewal and differentiation programs of SCs and eventual induced genotoxicity [18, 19]. Finally, it is necessary to obtain a good level of intracellular staining in order to gain information on the distribution of SCs inside host tissue. In this scenario, SiO_2_-NPs functionalized with fluorescent molecules have been extensively used for optical imaging, as enclosed dye molecules show a higher quantum yield and enhanced photostability respect to the same fluorophores freely dissolved in medium [20, 21].

Based on these assumptions, the rationale of the present study was to test safety, biocompatibility and feasibility to track SCs into tissue and organs of our lab-made amorphous fluorescent SiO_2_-NPs of uniform spherical size (50 ± 2 nm), do not bearing any capping agent and exposing at their surface only hydroxyl groups (silanols) and siloxane bridges [22]. In view of this purpose, in vitro analysis of labelled human bone marrow-derived MSCs (hMSCs) was assessed to deepen the aspect of toxicity and alteration of mesenchymal properties eventually related to long-term interaction of SiO_2_-NPs with these cells. Moreover, hMSCs labelled with fluorescent SiO_2_-NPs were injected in ex vivo perfused rat hearts to investigate their distribution and engraftment inside ventricular tissue.

## Results and discussion

### *In vitro* evaluation of the biosafety of 50 nm SiO_2_-NPs on hMSCs

#### hMSCs exposed for 16 h–50 nm SiO_2_-NPs 50 µg·mL^−1^ display good labeling, enclose SiO_2_-NPs inside lysosomes and are not subjected to oxidative stress

**Fig. 1:**
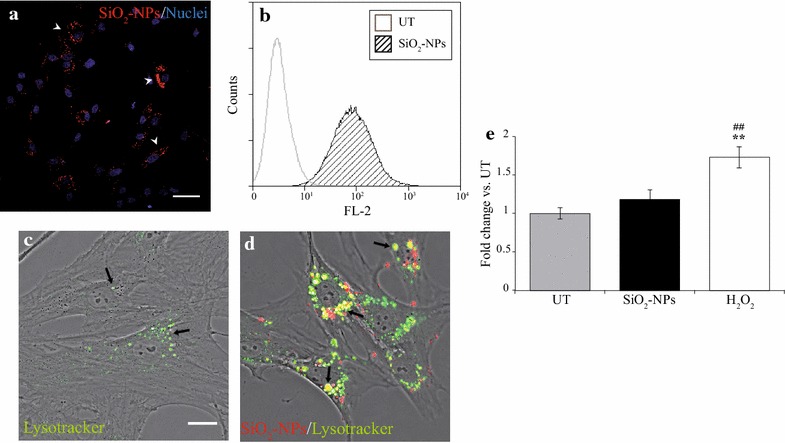
16 h exposure of hMSCs to 50 nm SiO_2_-NPs 50 µg·mL^−1^: cell labelling, intracellular localization and ROS production. **a** Staining of hMSCs with SiO_2_-NPs (*red*) after 16 h (t0). Nuclei were counterstained with Hoechst-33342 (*blue*). Magnification 40×, scale bar 50 µm. **b** Representative flow cytometry histogram of percentage hMSCs labelled with SiO_2_-NPs at t0. Untreated (UT) cells (*white histogram*) result negative for red fluorescence (FL-2), whereas labelled cells (SiO_2_-NPs, *hatched histogram*) emit strong red fluorescence due to internalized SiO_2_-NPs. **c**, **d** Confocal acquisitions of UT- (**c**) and SiO_2_-NPs-treated (**d**) hMSCs at t0 labelled with Lysotracker Green to reveal lysosomal compartments. Co-localization between fluorescent SiO_2_-NPs (*red*) and Lysotracker (*green*) was displayed by the system as yellow. Magnification 63×, scale bar 20 µm. **e** ROS production at t0 evaluated with DCFH-DA assay. hMSCs treated for 2 h with H_2_O_2_ 600 µmol L^−1^ served as positive control. Fluorescent emission from each sample was normalized by the mean UT value. ** p < 0.01 vs. UT and ## p < 0.01 vs. SiO_2_-NPs

Previous works from our laboratory documented that 50 nm SiO_2_-NPs at the dose of 20 µg·mL^−1^ were taken by hMSCs via active endocytosis, stored inside late endosomes and lysosomes and maintained elevated photostability at the acidic pH typical of these organelles [23, 24]. However, for the final purpose of visualizing labelled cells inside rat hearts increased dose and incubation time need to be tested, due to the small magnification necessary to appreciate the cells inside the whole tissue. Indeed, in the present study confocal analysis after 16 h exposure of hMSCs to the dose of 50 µg·mL^−1^ (here referred as t0) produced appropriate and bright fluorescent staining (Fig. 1a, arrowheads). Correspondingly, flow cytometry analysis (Fig. 1b) revealed that the mean number of labelled cells was 95.78 ± 1.27 %, indicating that 50 nm SiO_2_-NPs at the dose of 50 µg·mL^−1^are optimal contrast agents for hMSCs. Furthermore, despite the different dose and incubation time, confocal analysis of SiO_2_-NPs-exposed hMSCs labelled with Lysotracker Green confirmed that these NPs were stored inside lysosomes (Fig. 1d), according with the findings obtained in our earlier study [23].

Nevertheless, marked differences in lysosomal morphology and distribution were observed between untreated (UT) hMSCs and cells exposed to SiO_2_-NPs, as the former (UT cells) presented few and small lysosomes prevalently localized around nuclei (Fig. 1c, arrows), whereas the latter (SiO_2_-NPs-treated cells) displayed an increase of these organelles. Indeed, lysosomes were higher in number and assumed the aspect of vacuoles covering a greater portion of the cell body (Fig. 1d, arrows). These findings were in agreement with a recent work showing that 50 nm SiO_2_-NPs induced high vacuolization of human cerebral endothelial cells due to marked autophagic response as well as lysosomal involvement [25]. Interestingly, elevated reactive oxygen species (ROS) levels have been reported as one of the most important mechanisms of toxicity mediated by internalized SiO_2_-NPs in several cell types [17, 26], therefore, associated autophagy might be activated to limit ROS-dependent damage of cellular structures [27]. However, in the present study internalized SiO_2_-NPs did not induce any appreciable increase of ROS in hMSCs at t0 (Fig. 1e). This apparent discrepancy with literature data might be due to the fact that bone marrow-derived MSCs are highly resistant to oxidative damage due to the well-known constitutive expression of the biochemical machinery to scavenge ROS [28]; more likely, under oxidative conditions these cells can activate a defensive system probably dependent on marked increase of endosomal activity [29, 30].

Taken together, these data suggest that after SiO_2_-NPs internalization hMSCs might undergo elevated lysosomal involvement in the absence of an evident ROS stress.

#### Internalized SiO_2_-NPs are not associated to cyto- and genotoxic stress in cultured hMSCs and do not alter their proliferative potential

**Fig. 2:**
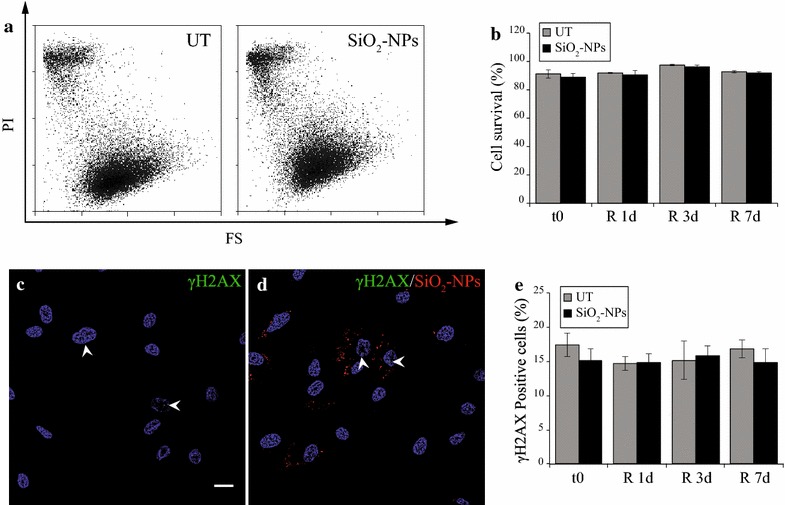
Assessment of cytotoxic and genotoxic stress in cultured hMSCs. **a** Representative flow cytometry histograms of UT- and SiO_2_-NPs -treated hMSCs at t0: forward scatter (FS) in function of propidium iodide fluorescence (PI) allows discriminating living cells (*bottom* population) from dead cells (*top* population). **b** Quantification of hMSCs viability during in vitro culture, represented as percentage variation respect to UT cells at the same time point. **c**, **d** Representative immunofluorescence experiments for γH2AX in UT- and SiO_2_-NPs -treated hMSCs at t0. Magnification 63×, scale bar 20 µm. **e** Quantification of percentage positive cells for nuclear γH2AX foci during culture

Demonstrated the absence of a detectable SiO_2_-NPs -induced oxidative stress at t0, the possibility of long-term toxicity was then investigated by analysing cell survival during prolonged in vitro culture (7 days) via propidium iodide (PI) staining (Fig. 2a, b). UT- and SiO_2_-NPs -treated hMSCs displayed a similar small amount of dead cells, as can be seen in the representative flow cytometry histograms at t0 (Fig. 2a, top populations). Quantification of percentage cell survival then revealed the same trend also after 1 (R 1d), 3 (R 3d) and 7 (R 7d) days of recovery in standard medium (Fig. 2b), thereby indicating that SiO_2_-NPs did not lead to significant cytotoxicity in hMSCs both after their uptake and at later time points of in vitro culture.

Another sign of SiO_2_-NPs-dependent toxicity might be their interaction with components of the nucleus or the genome itself. However, to date there are still limited and contrasting evidences in literature about genotoxicity, likely depending on the different cellular models, silica NPs types and/or concentrations used [31, 32]. In the present study, reconstructions of confocal acquisitions along the Z-axis indicated that SiO_2_-NPs did not localize inside nuclei during the time course of in vitro culture (Additional file 1 Panels A–D), thereby excluding a direct interaction between these NPs and the cell genome. Furthermore, immunofluorescence experiments of the phosphorylation at Ser^136^ of histone H2AX (γH2AX) (Fig. 2c, d), a specific marker of DNA double-strand-breaks (DSBs) [33], showed that at t0 both UT- and SiO_2_-NPs-hMSCs displayed very few γH2AX foci respect to irradiation at 10 Gy, which was used as a positive control for the reaction (Additional file 1 Panel E, arrows). Subsequent quantification highlighted that positive cells for γH2AX foci varied between 14.72 ± 1.04 and 16.78 ± 1.3 % for UT-hMSCs and between 14.85 ± 1.96 and 15.83 ± 1.47 % for SiO_2_-NPs-cells (Fig. 2g), with a comparable mean number of foci between the two conditions (Additional file 1 Panel F), thus demonstrating that cultured hMSCs did not undergo basal increase of DSBs. Altogether, present data confirm that 16 h exposure to 50 nm SiO_2_-NPs at the dose of 50 µg·mL^−1^ is safe and not associated to cyto- or genotoxic effects in hMSCs. This is also in line with the low ROS level observed at t0 (Fig. 1e).

**Fig. 3:**
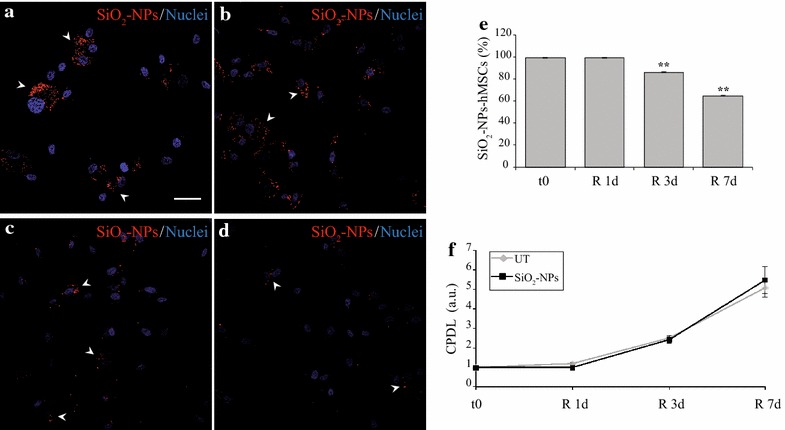
SiO_2_-NPs persistence in cultured hMSCs and assessment of their proliferation potential. **a–d** Distribution of SiO_2_-NPs (*red*) in hMSCs at t0 (**a**), R 1d (**b**), R 3d (**c**) and R 7d (**d**) time points. Nuclei were counterstained with Hoechst-33342 (*blue*). Magnification 40×, scale bar 50 µm. **e** Quantification of positive cells respect to total populations considered during confocal analysis. ** p < 0.01 vs. t0. **f** Proliferation rate of UT- and SiO_2_-NPs -treated hMSCs. For each time point, cells were counted to determine the “Cumulative Population Doubling Level” (CPDL) as an index of proliferation. Values for t0 were normalized to 1. a.u.: arbitral units

Finally, we analyzed the impact of SiO_2_-NPs on the proliferative potential of these cells. Representative immunofluorescence images show that at t0 and R 1d the majority of SiO_2_-NPs -treated hMSCs displayed considerable amounts of intracellular red fluorescence due to the presence of SiO_2_-NPs (Fig. 3a, b). However, at R 3d and R 7d time points a progressive decrease of fluorescent NPs per cell was observed (Fig. 3c, d). Quantification of these experiments as percentage labelled cells respect to the total population (Fig. 3e) revealed that the mean value at t0 was 99.22 ± 0.07 %, in accordance with flow cytometry results (Fig. 1b). Furthermore, a significant decrease at R 3d and R 7d was observed (both p < 0.01 vs. t0), with 64.86 ± 0.51 % of labelled cells at R 7d. Remarkably, co-localization with lysosomes was observed throughout the time course of in vitro cell culture (mean Pearson’s R values: 0.671 ± 0.01 for t0, 0.670 ± 0.02 for R1d, 0.693 ± 0.02 for R3d and 0.652 ± 0.04 for R7d), despite progressive decrease of internalized NPs per cell (Additional file 2 Panels A–D, arrows).

It is plausible that time-dependent reduction of fluorescent SiO_2_-NPs inside hMSCs is due to their redistribution into newly formed/daughter cells. In fact, the in vitro proliferation rate of hMSCs was not affected by internalized SiO_2_-NPs (Fig. 3f), in particular a gradual increase of cumulative population doubling levels (CPDL) was observed, reaching at R 7d 5.06 ± 0.46 for UT- and 5.45 ± 0.7 for SiO_2_-NPs-treated cells. As a further confirmation, the time-dependent decline of intracellular NPs was markedly inhibited when labelled hMSCs were cultured in complete DMEM supplemented with 1 % FBS, a specific condition that minimize cell divisions during culture (Additional file 2 Panels E–F). These data are in agreement with previous studies showing a dilution of loaded SiO_2_-NPs upon cell division in both human adenocarcinomic lung epithelial and murine pre-osteoblast cells [34–36].

Taken together, our results confirm that SiO_2_-NPs had no impact on the typical proliferation rate of hMSCs.

#### Treatment with SiO_2_-NPs s does not modify stemness properties of cultured hMSCs

Another possible influence of SiO_2_-NPs on hMSCs function might be the alteration of their stemness. In particular, hMSCs are characterized by the expression of a well-known set of surface antigens and by the ability to differentiate into chondrocytes, adipocytes and osteoblasts and to commit to other cell lineages, among them neurons and cardiomyocytes [37–40].

**Fig. 4:**
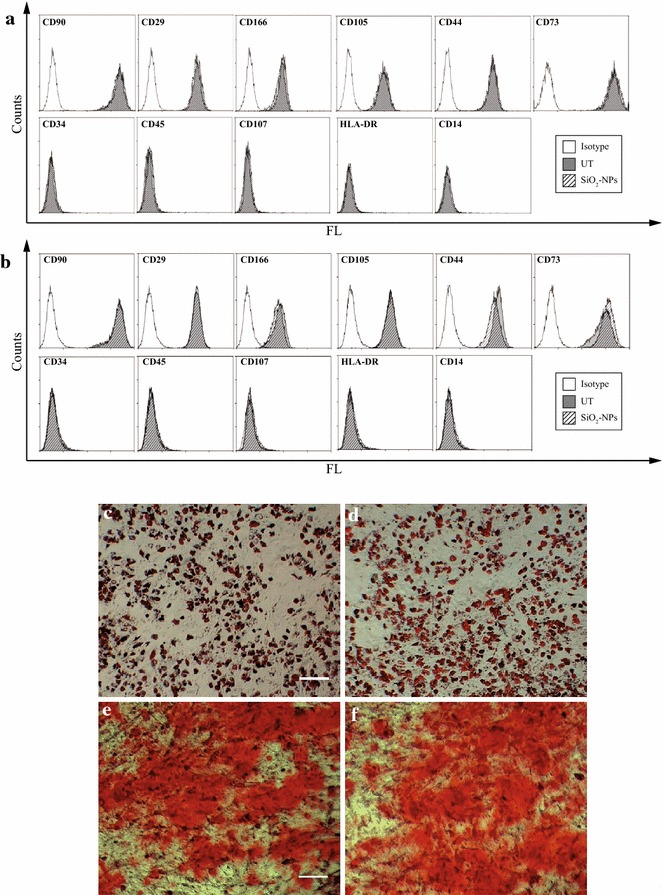
Assessment of stemness and differentiation potential in cultured hMSCs. Immunophenotypic characterization of UT and SiO_2_-NPs-treated hMSCs at t0 **(a)** and R 7d **(b)**. An anti-isotype IgG was used as negative control for the reaction. **c**, **d** Representative images of UT (**c**) and SiO_2_-NPs (**d**) cells after 18 days of adipose differentiation. Lipid vacuoles are stained with Oil Red. Magnification 10×, scale bar 100 µm. **(e**, **f)** Representative pictures of UT- (**e**) and SiO_2_-NPs -exposed (**f**) hMSCs after 21 days of osteogenic differentiation. Alizarin Red was used to reveal extracellular deposition of calcium salts. Magnification 10×, scale bar 100 µm

In this study, flow cytometry analysis of a specific set of surface antigens on both UT- and SiO_2_-NPs -treated hMSCs showed that at t0 and at R 7d (Fig. 4a, b) internalized SiO_2_-NPs did not change the expression of the specific markers CD90, CD29, CD166, CD105, CD44 and CD73. Moreover, labelled hMSCs remained negative for CD34, CD45 and CD107, HLA-DR and CD14 surface molecules. Analogous results were obtained at R 1d and R 3d time points (data not shown).

Importantly, the differentiation potential of hMSCs was not affected by their exposure to 50 nm SiO_2_-NPs; indeed, cell differentiation towards the adipogenic lineage was identical between UT- and SiO_2_-NPs-exposed cells, as both experimental groups acquired the round morphology typical of adipocytes (Additional file 3, Panels A–B, arrows) and produced a similar amount of specific intracellular lipid deposits stained with Oil red (Fig. 4c, d). Also osteogenic differentiation confirmed absence of differences between UT- and treated hMSCs, which at terminal induction resulted completely covered by a dense extracellular matrix typical of osteocytes (Additional file 3 Panels C–D) mostly made up of calcium deposits stained by Alizarin red (Fig. 4e, f).

Hence, these results suggest that intracellular accumulation of SiO_2_-NPs did not influence the stemness features of hMSCs, in line with previous data with the same SiO_2_-NPs given at lower dose and incubation time [23]. Finally, together with lack of cytotoxicity and unaltered proliferation rate during culture, these findings represent an important step-forward in demonstrating the biosafety of these amorphous NPs as suitable contrast agents for in vitro labeling of MSCs, in a perspective of in vivo application.

### Feasibility of 50 nm SiO_2_-NPs to track hMSCs inside the heart

**Fig. 5:**
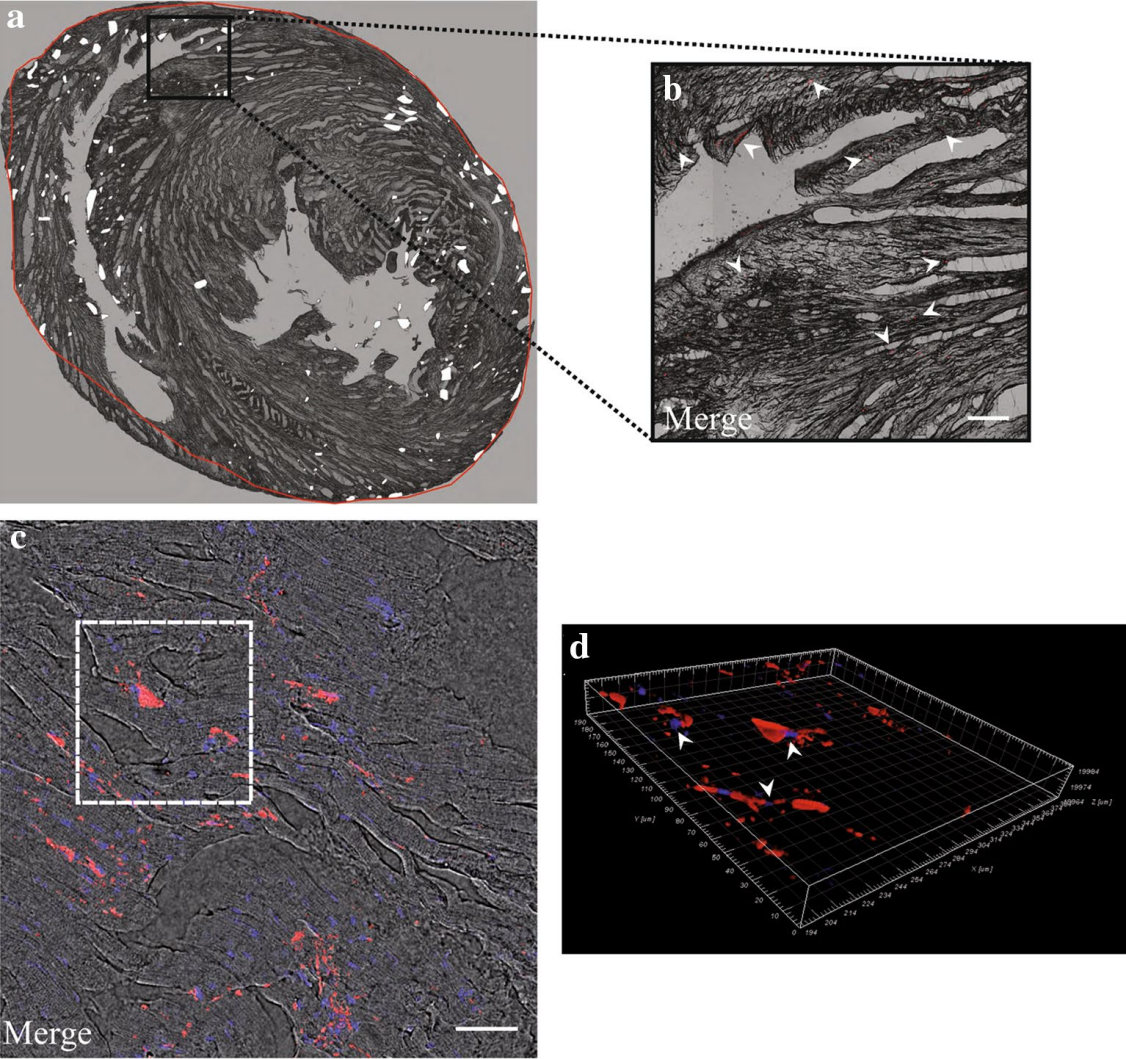
Imaging of SiO_2_-NPs -labelled hMSCs in normally perfused rat hearts. **a** Reconstruction of a 10 µm transverse slice (juxtaposition of consequent stacks of 5× magnification) to show the localization of labelled hMSCs in normal ventricles. *White points*: clusters of hMSCs. *Red line*: perimeter of cell distribution inside normal ventricles. **b** Subset showing spare distribution of labelled hMSCs (*red*) inside cardiac tissue. Magnification 5×, scale bar 200 µm. **c** Representative confocal reconstruction with superposition of bright field, hMSCs (*red*) and nuclei (*blue*). The *white dotted line* limits the perimeter of the volume illustration showed in **(d)**, where *arrowheads* stress the co-localization between labelled hMSCs (*red*) and nuclei (*blue*)

**Fig. 6:**
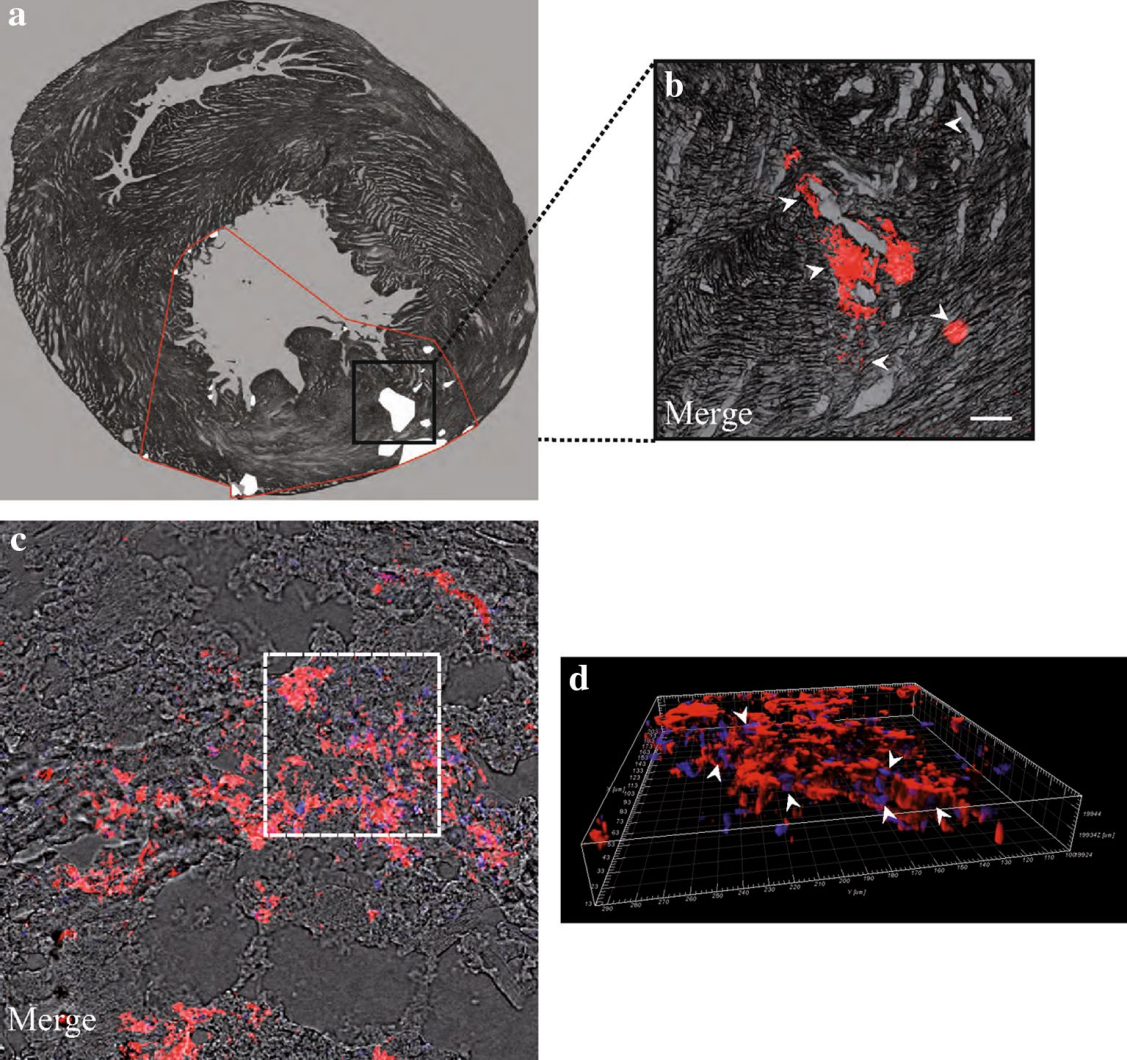
Imaging of SiO_2_-NPs -labelled hMSCs inside infarcted hearts. **a** Reconstruction of a 10 µm transverse slice to show the distribution of labelled hMSCs in infarcted hearts. *White points*: clusters of hMSCs. *Red line*: perimeter of cell distribution inside infarcted ventricles. **b** Subset illustrating labelled hMSCs (*red*) clustered inside ventricular tissue. Magnification 5×, scale bar 200 µm. **c** Representative confocal reconstruction with superposition of bright field, hMSCs (*red*) and nuclei (*blue*). The *white dotted line* is the perimeter of the volume representation in **(d)** to underline the higher co-localization between labelled hMSCs (*red*) and nuclei (*blue*) due to major cell aggregation typical of the injured area. Magnification 40×, scale bar 50 µm

After the profound characterization of the impact of 50 nm SiO_2_-NPs on viability, proliferation and stemness properties of in vitro cultured hMSCs, a further important aim of the present study was to ascertain the traceability of injected SCs in the challenging condition of a beating heart, subjected or not to ischemia/reperfusion (I/R). To this aim an ex vivo model of perfused rat heart was used. Cells employed for these investigations were harvested at t0, as this time point allowed obtaining almost the totality of the cells stained with 50 nm SiO_2_-NPs with no interference of culture-dependent dilution of internalized NPs, seen above (Fig. 3). In non-ischemic hearts, reconstruction of a representative transverse section highlighted that labelled cells, injected into the myocardium apex, were widespread distributed inside the walls of both right and left ventricles (Fig. 5a). Focus on a specific low magnification field underlined that hMSCs were clearly labelled by internalized fluorescent SiO_2_-NPs (Fig. 5b, arrowheads). In addition, confocal analysis at higher magnification (Fig. 5c) and volume reconstruction of a particular area of the same field (Fig. 5d, arrowheads) confirmed that cells were roughly dispersed inside the tissue, as often co-localization of one nucleus per labelled cell was observed. Concerning infarcted hearts, cells were injected into the apex 30 min after the ligation of the left descending coronary artery (LDCA) was removed. In these conditions, the pattern of cell distribution within the left ventricle myocardium depicted a major concentration of hMSCs close to the I/R lesion (Fig. 6a, white points) and the bright red fluorescence emitted by internalized SiO_2_-NPs underlined larger cell clusters (Fig. 6b, arrowheads). Higher magnification analysis (Fig. 6c) and volume reconstruction of a specific field (Fig. 6d, arrowheads) then revealed a higher number of nuclei co-localizing with red fluorescent cells respect to normally perfused hearts.

Notably, the peculiar distribution of MSCs inside normal and infarcted hearts was already evidenced by our previous work in which also rat bone marrow-derived MSCs were spread in normal hearts and more aggregated inside injured areas [41]. Hence, this finding might be considered as another confirmation that internalized SiO_2_-NPs do not alter the overall phenotype of hMSCs.

**Fig. 7:**
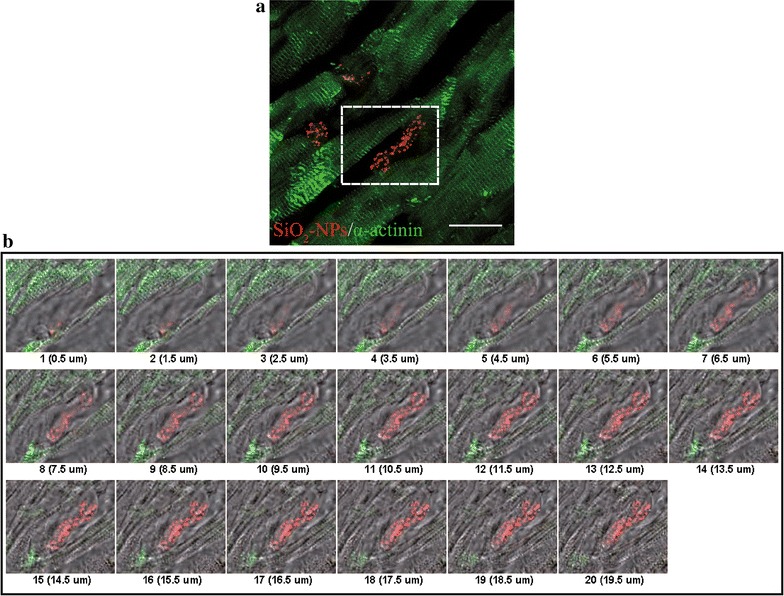
Ultrastructural analysis of labelled hMSCs engrafted inside ventricular tissue. Representative confocal reconstruction of a normal ventricle. **a** Superposition of sarcomeric α-actinin staining (*green*) and SiO_2_-NPs internalized in hMSCs (*red*). Magnification 100×, scale bar 20 µm. **b** Consequent slices along the Z-axis from the subset in A (*dashed* perimeter), to display morphological rearrangement of a hMSC between cardiac fibers; superposition of α-actinin (*green*), SiO_2_-NPs (*red*) and correspondent bright field to reveal cell body

Finally, confocal 100× analysis of a representative normal heart section was assessed to clarify the ultrastructure of labelled hMSCs inside heart tissue (Fig. 7). 100× magnification and staining of cardiac tissue with sarcomeric α-actinin allowed to better appreciate red fluorescent SiO_2_-NPs entrapped inside hMSCs (Fig. 7a). Nonetheless, superposition of the bright field in consequent slices along the Z-axis of a single hMSC revealed that aggregates of SiO_2_-NPs were spread along the entire thickness of the cell engrafted between cardiac fibres (Fig. 7b).

## Conclusions

Here we demonstrate that internalized 50 nm SiO_2_-NPs do not lead to long-term cyto- or genotoxic outcomes in cultured hMSCs, with concomitant preservation of stemness and proliferative properties of these cells. Moreover, findings on the beating hearts demonstrate that fluorescently labelled SiO_2_-NPs allow appropriate imaging and distribution of hMSCs in both normal and injured hearts.

A couple of methodological considerations need to be briefly outlined. First, one limitation of the isolated heart model might be the restricted time (in this case 6 h) offered before deterioration of the experimental preparation; however, for this study it resulted more accessible with respect to the in vivo counterpart, especially during simulation of I/R, due to excluded external interferences of neuro-hormonal responses, endothelial/neutrophil influence or massive inflammatory responses [41, 42]. Second, limited tissue penetrability of fluorescence might restrict the in vivo application of fluorescently labelled SiO_2_-NPs for SCs tracking. Yet, the ease of functionalization of these silica platforms and their biosafety might be exploited to entrap inside them contrast agents allowing more penetrating and non invasive imaging, such as magnetic resonance and ultrasound, which have already been applied in cardiac regenerative medicine [14, 43, 44].

Taken together, our protocol of 16 h exposure of hMSCs to 50 nm SiO_2_-NPs at the dose of 50 µg mL^−1^ possess powerful features of biosafety and compatibility that make these NPs suitable candidates for proper and potentially harmless labelling oriented to SCs tracking inside the heart. For the final purpose of obtaining long-term and non-invasive cell imaging in in vivo models, encapsulation of more penetrating contrast agents inside SiO_2_-NPs will enhance the applicability of these powerful core shells.

## Methods

### Drugs and SiO_2_-NPs

All drugs were purchased by Sigma, unless directly mentioned. Chemical composition and preparation of red fluorescent cyanine dye-doped SiO_2_-NPs “IRIS Dots” are detailed elsewhere [22]. Obtained SiO_2_-NPs exhibited a diameter of 50 ± 2 nm, possessed elevated morphologic homogeneity and displayed bright fluorescence emission and high photostability. An aliquot of pure SiO_2_-NPs was prepared without the addition of the fluorophore and used to evaluate ROS production, surface phenotype and differentiation potential of hMSCs.

### Culture and treatment of hMSCs

hMSCs isolated from the bone marrow of healthy donors were commercially obtained from Lonza (Lonza Group Ltd., Switzerland) and were used for experiments at passages 4–8. Briefly, hMSCs were cultured in DMEM supplemented with 1 % sodium pyruvate, 1 % nonessential amino acids, 1 % kanamycin, 1 % l-glutamine, 0.1 % β-mercaptoethanol (complete DMEM) and 10 % fetal bovine serum (FBS, Euroclone, Italy) (standard medium) and kept in an atmosphere of 5 % CO_2_, 95 % air at 37 °C in a humidified incubator. After reaching 80 % confluence, cells were detached using 0.25 % trypsin-ethylenediamine-tetra acetate (EDTA) 1 mmol·L^−1^, counted with a Neubauer chamber, seeded at the density of 6500 cell cm^−2^, given 24 h to settle and then incubated with the two following protocols: (a) *SiO*_*2*_-*NPs*: cells were exposed for 16 h with a suspension of water dissolved SiO_2_-NPs 50 µg·mL^−1^ in complete DMEM/1 % FBS; (b) *Untreated (UT)*: cells were incubated for 16 h with complete DMEM/1 % FBS supplemented with the same volume of sterile H_2_O in which SiO_2_-NPs were dispersed. Subsequently, samples of both conditions were washed twice with warm phosphate-buffered saline (PBS, Euroclone) and analysed after the treatment (t0) or after 1 (R 1d), 3 (R 3d) and 7 days (R 7d) of in vitro recovery in standard medium.

### Confocal microscopy

The inverted confocal laser scanning microscope LSM 510 (488 and 568 nm excitation wavelengths, Carl Zeiss, Germany) equipped with 5× (N.A. 0.15), 63× (N.A. 1.40, oil immersion) and 100× (N.A. 1.13, oil immersion) objectives was used to obtain XY images or stacks along the Z-axis without nuclei counterstaining (co-localization between SiO_2_-NPs and lysosomes; reconstruction of whole ventricular sections; ultrastructural analysis of heart samples). The TCS SP5 confocal laser microscopy system (Diode 405, Argon and He–Ne lasers, Leica Microsystem S.r.l., Italy) equipped with 40× (N.A. 1.25, oil immersion) and 63× (N.A. 1.40, oil immersion) objectives was used to obtain stacks along the Z-axis with nuclei counterstaining (labelling rate, intracellular persistence and interaction of SiO_2_-NPs with nuclei in cultured hMSCs; detection of γH2AX nuclear foci in cultured hMSCs; 40× analysis of ventricular sections).

Representative images presented in this study were processed with: ImageJ^®^ (Rasband, W.S., ImageJ, U.S. National Institutes of Health, Bethesda, MD, USA, http://www.rsb.info.nih.gov/ij/, 1997–2015) for reconstructions along the Z-axis of confocal stacks and for juxtapositions of XY images (5x magnification) to obtain whole transverse sections; Imaris (BitPlane AG, version 7.2.3) for volume representations of SiO_2_-NPs interaction with nuclei and heart tissue sections (*Surpass* viewer) and for representation of consequential slices (*Gallery* viewer).

### hMSCs labelling with SiO_2_-NPs and analysis of ROS production

hMSCs treated onto glass coverslips were washed twice with PBS and fixed for 15 min room temperature (r.t.) with cold 4 % paraformaldehyde (PAF) in PBS (pH 7.3). After nuclear counterstaining with Hoechst-33342 5 µg mL^−1^ for 15 min r.t, coverslips were mounted onto glass slides with Mowiol (Calbiochem, USA) and conserved at 4 °C. The labelling rate at t0 was quantified by flow cytometry: both UT and SiO_2_-NPs -treated cells were harvested, collected with a CyAN ADP flow cytometer (at least 30,000 events per sample) and analysed with Summit 4.3 software (Beckman Coulter, USA). Autofluorescence of UT cells was previously subtracted from the analysis, data were presented in a histogram of number of events (Counts) vs. FL-2 Log (FL-2) and labelled cells were quantified as percentage of the total population.

To mark late endosomes and lysosomes, cells treated onto glass-bottomed dishes (MatTeck, USA) were incubated 15 min at 37° C with the fluorescent dye LysoTracker Green 2 µmol L^−1^ (Life Technologies, Italy) in complete DMEM, washed with sterile PBS and analysed with confocal microscopy. For each time point, quantification of co-localization was expressed as mean Pearson’s R value, calculated with the ImageJ^®^ tool *“Co*-*localization finder”*.

To evaluate ROS production in hMSCs, cells seeded in Nunc™ black flat-bottomed 96-wells (Thermo Fisher Scientific, USA) underwent UT or SiO_2_-NPs protocols, whereas treatment for 2 h with or hydrogen peroxide (H_2_O_2_) 600 µmol L^−1 ^ was used as a positive control for induction of oxidative stress. Samples were washed with PBS and incubated for 30 min at 37 °C in the dark with the specific probe Di Chloro-dihydro-Fluorescein Di Acetate (DCFH-DA) 10 µmol L^−1^ dissolved in standard medium w/o phenol red. Fluorescence emission (excitation filter: 485 ± 20 nm; emission filter: 535 ± 25 nm) was read with an Infinite F200 microplate reader (Tecan Group Ltd., Switzerland) and data from each sample were normalized on UT value.

### SiO_2_-NPs-dependent cyto- and genotoxicity

hMSCs survival was evaluated by collecting both detached and adherent cells to incubate them for 5 min r.t. in the dark with a solution of propidium iodide (PI) 1 µg·mL^−1^ in cold PBS. At least 30,000 events per sample were collected with a CyAN ADP flow cytometer, viable cells were evaluated on FL-2 Log (PI) vs. Forward Scatter Lin (FS) plots and expressed as percentage of living cells respect to the relative UT time-point.

Genotoxic stress was analysed by detecting nuclear foci of the histone γH2AX. hMSCs 1 h after 10 Gy irradiation were used as a positive control for the reaction. Briefly, cells treated onto glass coverslips were washed with PBS, fixed for 15 min with cold 4 % PAF, permeabilized for 15 min with 0.1 % Triton X-100 and blocked for 30 min with a solution of 6 % wt/vol bovine serine albumin (BSA) and 2.5 % normal goat serum (NGS) in PBS (all at r.t.). Incubation for 3 h at 4 °C with the primary antibody mouse anti-phospho H2AX^Ser136^ (γH2AX, clone JBW301, Millipore, USA) 1:500 in PBS was followed by staining for 30 min at 37 °C with the secondary antibody anti-mouse Alexa Fluor 488 (Life Technologies) 1:500 in PBS. After nuclear staining for 15 min r.t. with Hoechst-33342 5 µg mL^−1^, coverslips were mounted onto glass slides and conserved at 4 °C. Quantification of these experiments was assessed with confocal microscopy by random counting at least 250 cells per sample to obtain the per cent number of cells with nuclear foci (γH2AX positive cells) respect to the total considered population.

### SiO_2_-NPs persistence inside hMSCs and assessment of proliferation potential

The time course of SiO_2_-NPs persistence inside hMSCs was evaluated on cells treated onto glass coverslips through immunofluorescence and confocal microscopy. Quantification was assessed with the same method used for γH2AX foci by random counting at least 250 cells per sample to obtain the per cent labelled cells (SiO_2_-NPs-hMSCs) respect to the total considered population.

Proliferation during in vitro culture was evaluated via cell counting: hMSCs treated into T25 flasks (Corning, USA) were harvested and counted with a Neubauer chamber considering 8 quadrants for each sample. Proliferation potential was expressed as “Cumulative Population Doubling Level” (CPDL) according to Yu et al. [45].

### Characterization of hMSCs surface phenotype

For each time point cells were harvested, washed with PBS supplemented with 1 % FBS and stained for 30 min at 4 °C with the following antibodies (1:10 in PBS, BD Pharmingen, USA): anti-CD90, anti-CD29, anti-CD166 (all conjugated with phycoerythrin PE); anti-CD105, anti-CD34, anti-CD45, anti-CD107, anti-HLA-DR, anti-CD14, anti-CD44 (all conjugated with fluorescein isothiocyanate FITC); anti-CD73 (conjugated with allophycocyanin APC, MiltenyiBiotec, Germany). Cells were then suspended in cold PBS, at least 30,000 events per sample were collected with CyAN ADP flow cytometer and subsequent histograms of number of events (Counts) vs. Fluorescence Log (FL) were obtained.

### In vitro adipogenic and osteogenic differentiation

After the treatments cells were seeded in 24-well plates at the density of 20,000 cells cm^−2^, given 24 h to settle and then treated for differentiation induction as previously described [46]. Briefly, adipose differentiation was induced with a specific adipogenic medium composed of standard medium supplemented with dexamethasone 1 µmol L^−1^, isobutylmethylxanthine (IBMX) 500 µmol L^−1^, indomethacin 100 µmol L^−1^and insulin 10 µg·mL^−1^. Medium was replaced twice a week until day 18, when lipid droplets were counterstained with Oil Red: samples at r.t. were washed with PBS, fixed 1 h with 10 % formalin neutral buffer solution, washed twice with distilled H_2_O, incubated 5 min with 60 % isopropanol and then treated 30 min with a solution of Oil Red 0.5 w/vol in 60 % isopropanol.

Osteogenic commitment was induced by means of a specific medium composed of standard medium supplemented with dexamethasone 100 nmol L^−1^, glycerol 2-phosphate 10 mmol L^−1^ and ascorbic acid 200 µmol L^−1^. Medium was changed twice a week until day 21, when extracellular calcium salts were revealed through Alizarin Red staining. Briefly, cells at r.t. were washed with PBS, fixed 1 h with 70 % ethanol, washed twice with distilled H_2_O and incubated 45 min with a solution of Alizarin Red 40 mmol L^−1^ (pH 4.2). For both assays, bright field images of morphological commitment at terminal differentiation were taken with a digital camera (Moticam 580) mounted on an optical inverted microscope (AE 2000, Motic, Spain) supplemented with 4× and 10× objectives. Representative images were then processed with ImageJ^®^.

### Perfusion of isolated rat hearts and injection of SiO_2_-NPs-hMSCs

This study conforms to the *Guide for the Care and Use of Laboratory Animals* published by the US National Institutes of Health (NIH Publication No. 85–23, revised 1996) and in accordance with the Italian ethical guidelines (L 96, 6 August 2013). The local ethical committee approved the research project. Experiments were performed on adult male Wistar rats (body-weight 450–550 g). Animals were heparinized (2500 U I.m., Roche, Italy) and anaesthetized with urethane (1 g/kg i.p.) 10 min later. The hearts were rapidly excised, cannulated via the aorta and retrogradely perfused with oxygenated Krebs-Henseleit buffer containing (in mmol·L^−1^) 127 NaCl, 17.7 NaHCO_3_, 5.1 KCl, 1.5 CaCl_2_, 1.26 MgCl_2_ and 11 d-glucose, supplemented with 5 µgm·L^−1^ lidocaine. A constant flow was adjusted with a proper pump (Watson-Marlow 313, UK) to obtain a typical coronary perfusion pressure of 80–85 mm Hg during initial stabilization. Thereafter, the same flow level (9 ± 1 mL·min^−1^g^−1^) was maintained throughout the experiment. Temperature of perfusate and hearts were kept constant at 37° C throughout the experiments.

Hearts were divided into two groups, normal and infarcted hearts, presented in Additional file 4. In normal hearts (Additional file 4 Panel A, n = 3), hMSCs were injected in the apex 90 min after the start of retrograde perfusion. In infarcted hearts (Additional file 4 Panel B, n = 4), after 30 min stabilization, the LDCA was occluded for 30 min (Additional file 4 Panel B, “Ischemia”, recognized by pale-coloured tissue after coronary occlusion and by a fall in left ventricular developed pressure) and then re-opened to full re-flow of the left ventricle (Additional file 4 Panel B, “Reperfusion”). hMSCs were injected in the apex after 30 min of re-oxygenation. All the experiments were stopped after total 6 h of retrograde perfusion, as this time was considered the end-point for proper ex vivo experiments, according to Penna et al. [41].

### Heart processing and immunofluorescence analysis of tissue slices

At the end of perfusion, atria and vasa were discarded and ventricles were fixed for 3 h r.t. with 4 % PAF with gentle stirring. Tissues were then washed with PBS, submerged in a solution of 30 % sucrose in PBS, allowed precipitating overnight at 4 °C and then incubated 30 min r.t. in a solution 1:1 of 30 % sucrose in PBS and TissueTek^®^ Optimal Cutting Temperature (O.C.T.™, Sakura FineTek, USA). Finally, tissues were embedded in O.C.T. and stored at −80 °C. 10 µm thick transverse slices were obtained starting from the apex with a CM 1900 cryostat (Leica Microsystem S.r.l.), placed onto Superfrost™ glass slides (Thermo Scientific, USA) and conserved at −20 °C. Sections used for ventricle reconstructions and 40× analyses were rinsed with PBS and, after nuclear staining for 15 min r.t. with Hoechst-33342 5 µg mL^−1^, they were mounted with Mowiol and conserved at 4 °C. Sections for ultrastructural analysis were processed for immunofluorescence. Briefly, they were rinsed in PBS, permeabilized for 20 min r.t. with 0.5 % Triton X-100 and blocked for 1 h r.t. with 6 % BSA and 2.5 % NGS in PBS. The primary antibody mouse anti-sarcomeric α-actinin 1:600 in PBS was incubated overnight 4 °C, whereas the secondary antibody anti-mouse Alexa Fluor 488 1:1000 in PBS was incubated for 1 h r.t. Finally, samples were mounted with Mowiol and conserved at 4 °C.

### Statistical Analysis

Data are expressed as mean ± standard error of the mean (S.E.M.) of at least three different experiments. Statistical comparisons were performed with Student’s *t* test or one-way analysis of variance (ANOVA) with Bonferroni correction. Differences with *p* ≤ 0.05 were regarded as statistically significant.

## Additional files

10.1186/s12951-015-0141-1 (A-D) hMSCs from t0 (A), R 1d (B), R 3d (C) and R 7d (D) show that SiO_2_-NPs (red) do not localize inside nuclei (blue). Volume reconstructions from 40x acquisitions. (E) 512 × 512 crop of a 63x acquisition to show γH2AX foci in hMSCs obtained 1 h after irradiation at 10 Gy (positive control). Scale bar 20 µm. (F) Mean number of γH2AX nuclear foci inside the population of positive cells considered for the analysis.
10.1186/s12951-015-0141-1 (A-D) Representative confocal acquisitions of living hMSCs with internalized SiO_2_-NPs (red) and lysosomes labelled with Lysotracker (green) at t0 (A), R 1d (B), R 3d (C) and R 7d (D) time points. Where there is co-localization, the system displays it as yellow merge (arrows). Magnification 63x, scale bar 20 µm. (E) Quantification of SiO_2_-NPs persistence inside hMSCs recovering in complete medium supplemented with 1 % FBS. (F) Proliferation rate of UT- and SiO_2_-NPs -treated hMSCs cultured in complete medium supplemented with 1 % FBS, expressed as CPDL. Values for t0 are represented as 1.
10.1186/s12951-015-0141-1 (A-B) Representative images of morphological changes in UT (A) and SiO_2_-NPs -treated (B) hMSCs after 18 days of induced adipose differentiation. Arrows underline the presence of lipid vacuoles inside round differentiated cells. (C-D) UT (C) and SiO_2_-NPs -treated (D) hMSCs after 21 days of osteogenic differentiation. Cells are clearly immersed in a dense extracellular matrix. Magnification 10x, scale bar 100 µm.
10.1186/s12951-015-0141-1 Experimental protocols for 6 h retrograde perfusion of isolated adult rat hearts. (A) Protocol applied for normal hearts. (B) Experimental procedure applied to simulate ventricular infarction via ligature of the LDCA (Ischemia 30 min) and its subsequent re-opening (Reperfusion). In both groups, SiO_2_-NPs -treated hMSCs were injected in the apex after 90 min from the start of retrograde perfusion.

## Abbreviations

SCs: stem cells
SiO_2_-NPs: silica nanoparticles
hMSCs: human bone marrow-derived mesenchymal stem cells
UT: untreated
ROS: reactive oxygen species
γH2AX: histone H2AX phosphorylated at Ser^139^
I/R: ischemia/reperfusion
FBS: fetal bovine serum
PBS: phosphate buffered saline
r.t.: room temperature
PAF: paraformaldehyde
