# Supplementary figures and images for: Human mesenchymal stem cells labelled with dye-loaded amorphous silica nanoparticles: long-term biosafety, stemness preservation and traceability in the beating heart
Source: J Nanobiotechnology. 2015 Oct 29;13:77. doi: 10.1186/s12951-015-0141-1 (PMC4625930; doi:10.1186/s12951-015-0141-1)

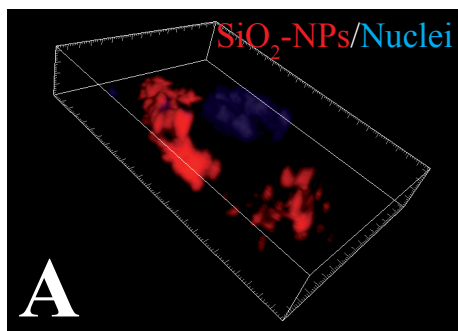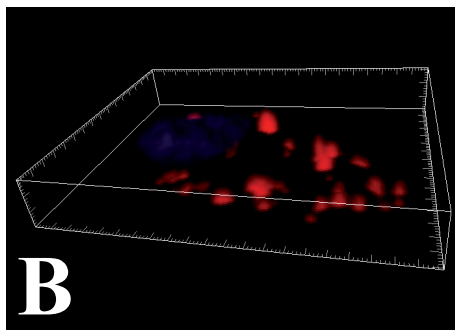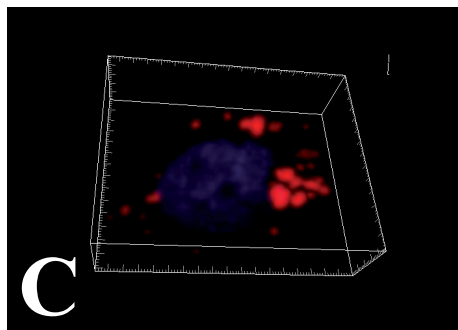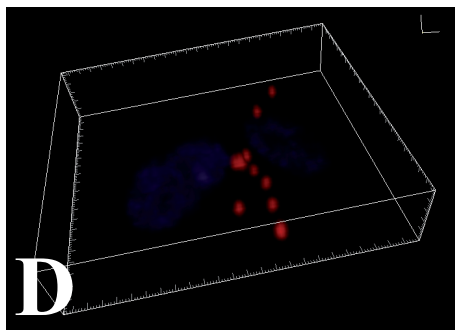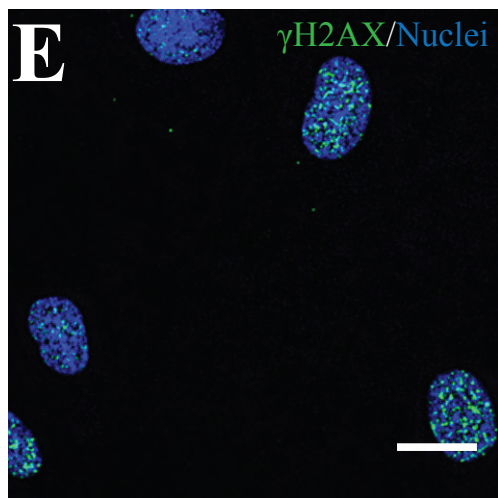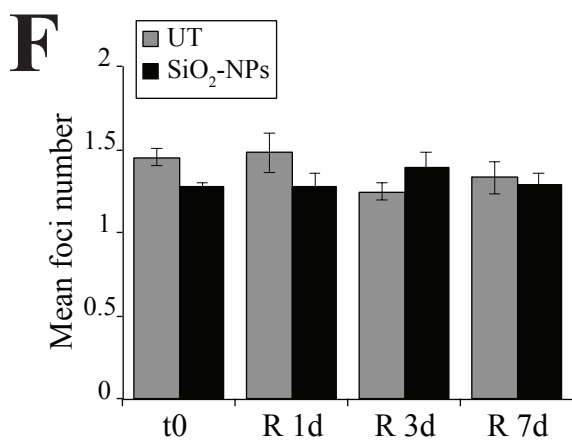

Supplement: Supplementary file 1 — 10.1186/s12951-015-0141-1 (A-D) hMSCs from t0 (A), R 1d (B), R 3d (C) and R 7d (D) show that SiO2-NPs (red) do not localize inside nuclei (blue). Volume reconstructions from 40x acquisitions. (E) 512 × 512 crop of a 63x acquisition to show γH2AX foci in hMSCs obtained 1 h after irradiation at 10 Gy (positive control). Scale bar 20 µm. (F) Mean number of γH2AX nuclear foci inside the population of positive cells considered for the analysis. [file 12951_2015_141_MOESM1_ESM.pdf]

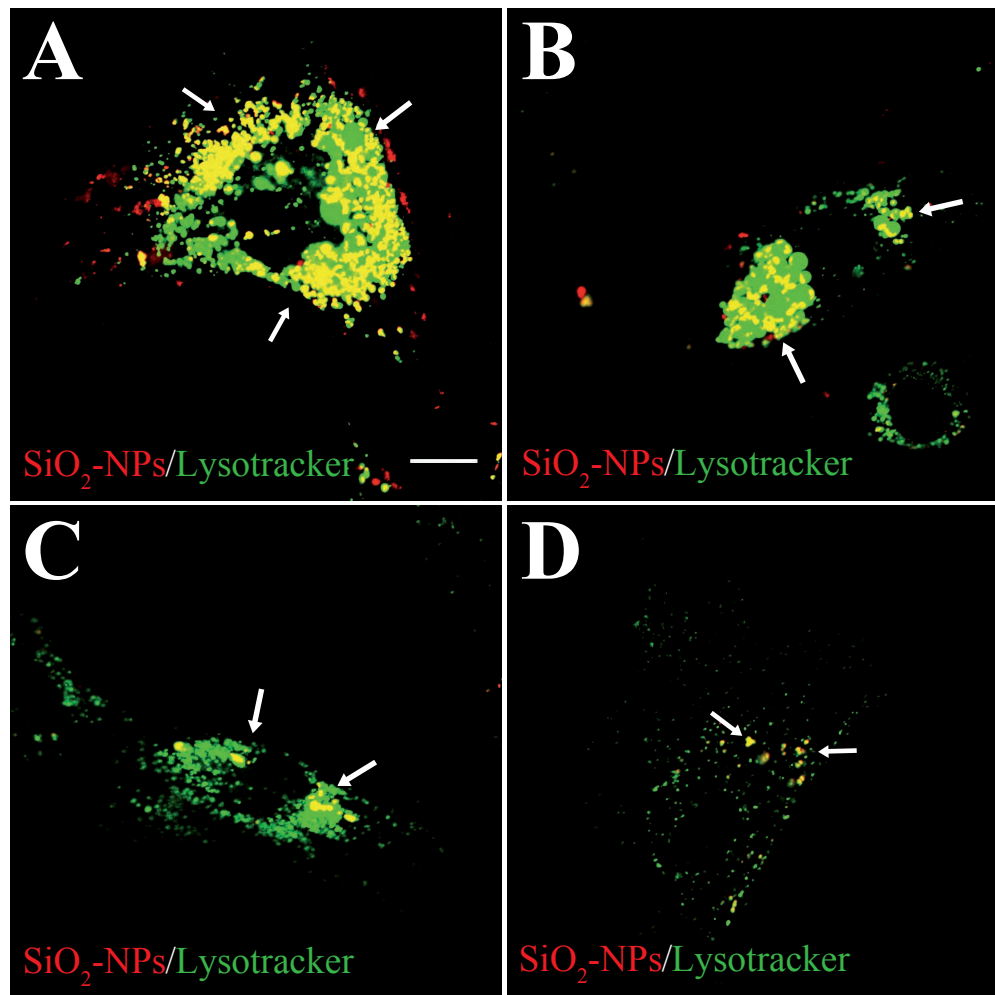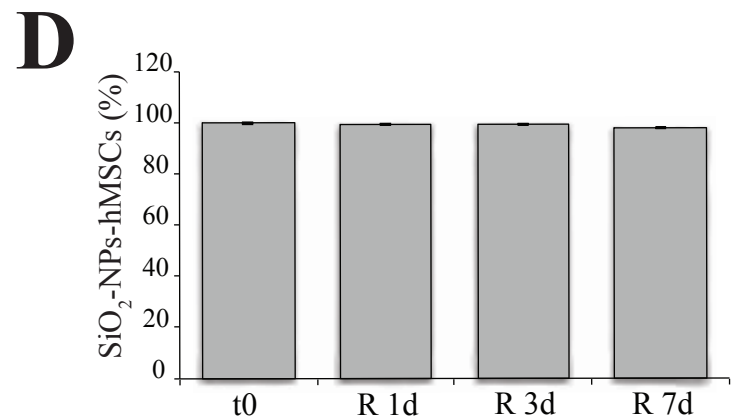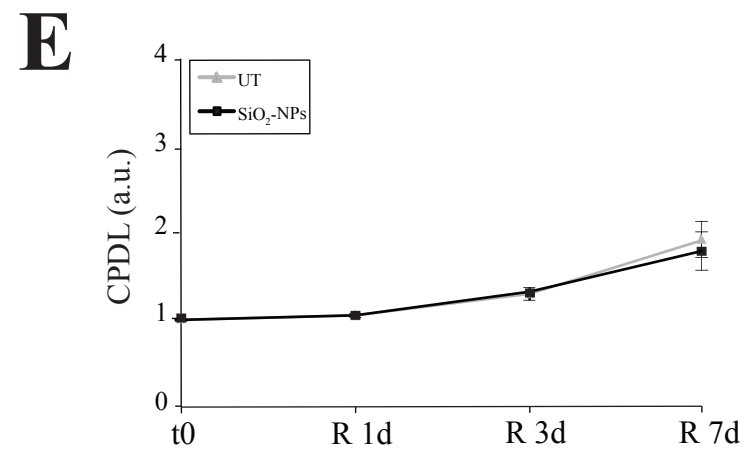

Supplement: Supplementary file 2 — 10.1186/s12951-015-0141-1 (A-D) Representative confocal acquisitions of living hMSCs with internalized SiO2-NPs (red) and lysosomes labelled with Lysotracker (green) at t0 (A), R 1d (B), R 3d (C) and R 7d (D) time points. Where there is co-localization, the system displays it as yellow merge (arrows). Magnification 63x, scale bar 20 µm. (E) Quantification of SiO2-NPs persistence inside hMSCs recovering in complete medium supplemented with 1 % FBS. (F) Proliferation rate of UT- and SiO2-NPs -treated hMSCs cultured in complete medium supplemented with 1 % FBS, expressed as CPDL. Values for t0 are represented as 1. [file 12951_2015_141_MOESM2_ESM.pdf]

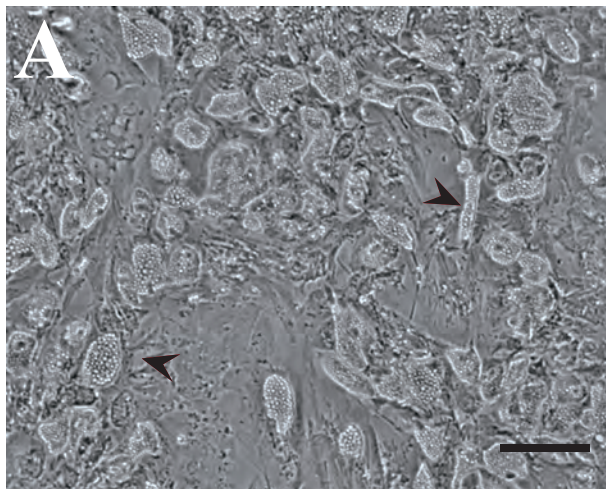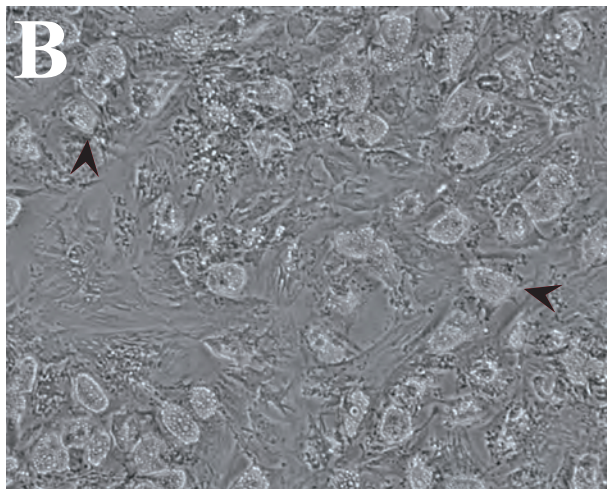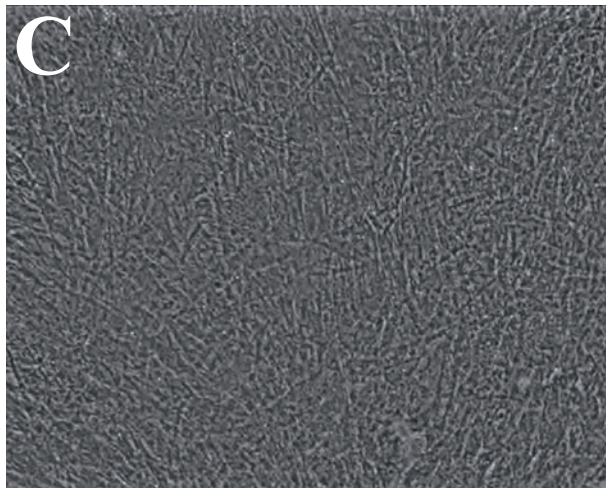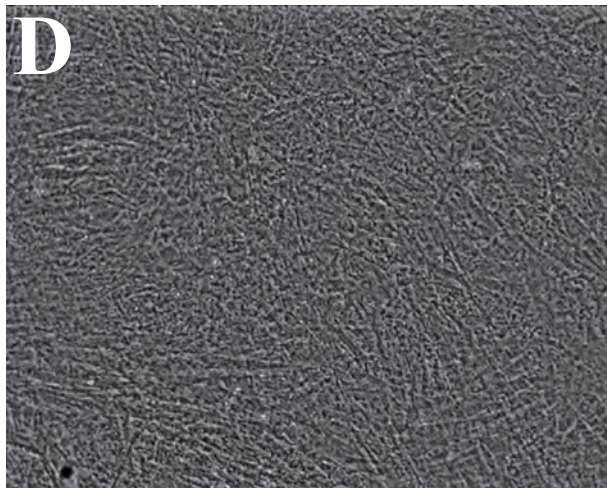

Supplement: Supplementary file 3 — 10.1186/s12951-015-0141-1 (A-B) Representative images of morphological changes in UT (A) and SiO2-NPs -treated (B) hMSCs after 18 days of induced adipose differentiation. Arrows underline the presence of lipid vacuoles inside round differentiated cells. (C-D) UT (C) and SiO2-NPs -treated (D) hMSCs after 21 days of osteogenic differentiation. Cells are clearly immersed in a dense extracellular matrix. Magnification 10x, scale bar 100 µm. [file 12951_2015_141_MOESM3_ESM.pdf]

**A**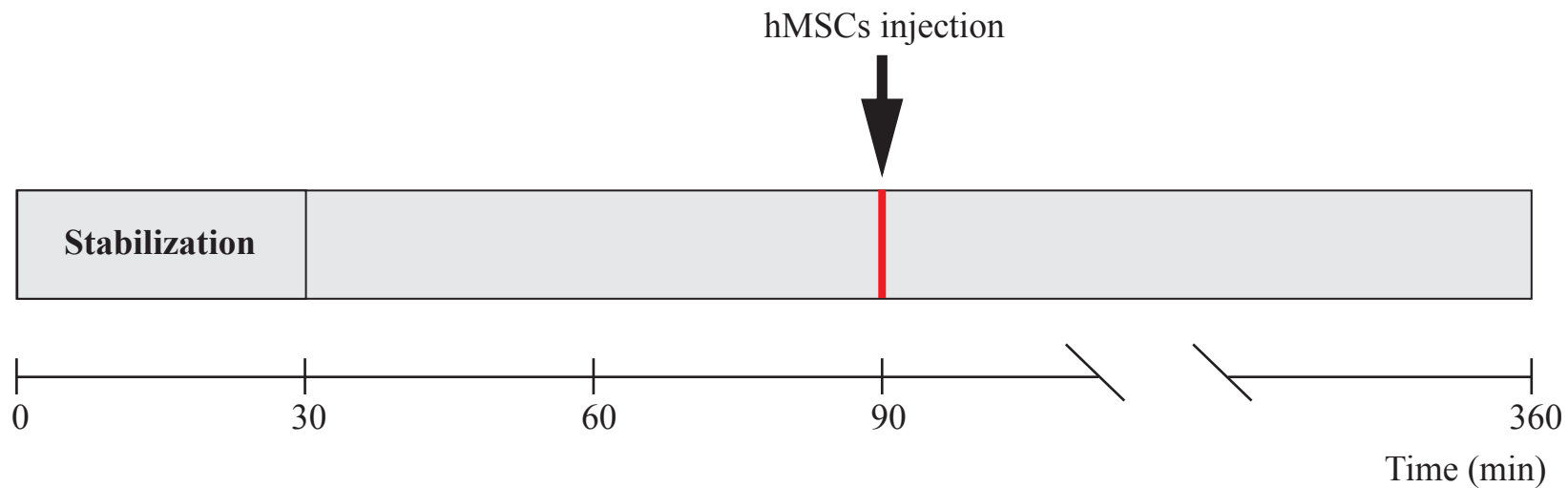**B**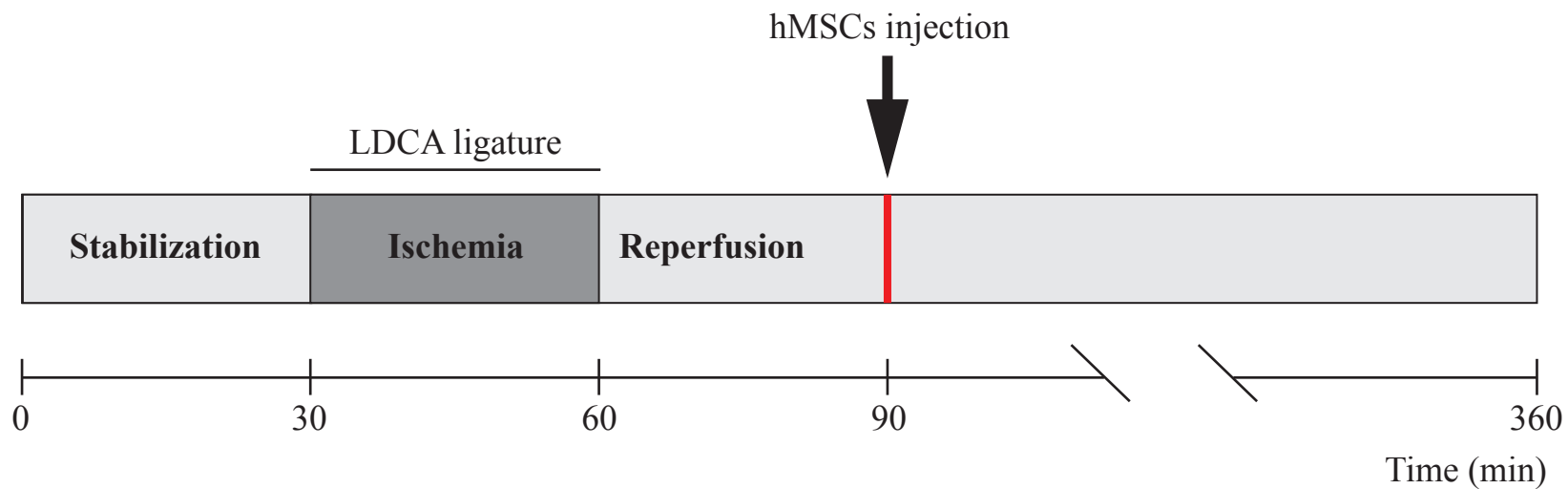

Supplement: Supplementary file 4 — 10.1186/s12951-015-0141-1 Experimental protocols for 6 h retrograde perfusion of isolated adult rat hearts. (A) Protocol applied for normal hearts. (B) Experimental procedure applied to simulate ventricular infarction via ligature of the LDCA (Ischemia 30 min) and its subsequent re-opening (Reperfusion). In both groups, SiO2-NPs -treated hMSCs were injected in the apex after 90 min from the start of retrograde perfusion. [file 12951_2015_141_MOESM4_ESM.pdf]
